# Explainable Machine Learning with Optimized Tree-Based Models and Statistical Validation for Data-Driven Wear Prediction of Carburized and Non-Carburized Engineering Steels

**DOI:** 10.3390/ma19194045

**Published:** 2026-09-22

**Authors:** S. R. Harisha, M. Arunadevi, B. R. N. Murthy, M. S. Srinath, Bilachi S. Ravindranath, Krishnamurthy D. Ambiger, Revanth N. Murthy

**Affiliations:** 1 Manipal Institute of Technology, Manipal Academy of Higher Education, Manipal 576104, India; harisha.sr@manipal.edu (S.R.H.); ravindranath.bs@manipal.edu (B.S.R.); krishnamurthy.mitmpl2022@learner.manipal.edu (K.D.A.); revanth.msismpl2025@learner.manipal.edu (R.N.M.); 2 Department of Mechanical Engineering, Ramaiah Institute of Technology, Bengaluru 560001, India; arunadevi.dsce@gmail.com; 3 Department of Mechanical Engineering, Dayananda Sagar College of Engineering, Bengaluru 560001, India; srinadme@gmail.com

**Keywords:** wear prediction, steel materials, decision tree, random forest, hyperparameter optimization, sensitivity analysis

## Abstract

**Highlights:**

**What are the main findings?**

- Dry sliding wear behavior of engineering steels was experimentally evaluated.
- Optimized Random Forest achieved superior wear prediction accuracy.
- SHAP analysis explained the influence of material and process variables.
- Statistical validation confirmed the robustness of the developed model.

**What are the implications of the main findings?**

- Provides an accurate and interpretable framework for wear prediction using optimized machine learning models.
- Reduces experimental time and cost through reliable data-driven wear estimation.
- Identifies dominant wear-governing parameters using SHAP-based explainable AI.
- Supports intelligent material selection, predictive maintenance, and digital manufacturing.

**Abstract:**

Accurate prediction of wear behavior in steel materials is essential for enhancing component durability and optimizing manufacturing processes. This study presents a comparative data-driven framework for wear prediction using Decision Tree (DT), Random Forest (RF), and a Physics-Informed Neural Network (PINN). Hyperparameter optimization for the DT and RF models was performed using GridSearchCV with 5-fold cross-validation to obtain optimal model configurations. Model performance was evaluated using the Root Mean Square Error (RMSE), Mean Absolute Error (MAE), and coefficient of determination (R^2^). In addition, sensitivity analysis was conducted by varying train–test split ratios, while residual analysis, confidence interval estimation, and SHAP-based feature importance analysis were employed to assess model reliability and interpretability. The Random Forest model achieved the best predictive performance on the test dataset with RMSE = 0.00495, MAE = 0.00273, and R^2^ = 0.7244, followed by the Decision Tree model with RMSE = 0.00565, MAE = 0.00275, and R^2^ = 0.6406. The implemented Physics-Informed Neural Network (PINN), incorporating a load–time physics constraint, exhibited significantly poorer performance, yielding RMSE = 0.15872, MAE = 0.12599, and R^2^ = −282.62, indicating that the available experimental dataset and simplified physics constraint were insufficient for effective physics-informed learning. SHAP analysis identified surface condition, material type, sliding time, and the load–time interaction as the most influential parameters governing wear behavior. The findings demonstrate that optimized tree-based machine learning models, particularly the Random Forest model, provide accurate, interpretable, and computationally efficient predictions for wear estimation in the investigated steel materials.

## 1. Introduction

Wear is a crucial failure mechanism that significantly impacts the performance and durability of engineering components operating under sliding or rolling contact [1]. Material loss due to wear not only reduces dimensional accuracy but also decreases mechanical efficiency, increases energy consumption, and shortens the service life of machinery. The economic impact of wear is substantial across various industries, including automotive, aerospace, mining, manufacturing, and power generation, where the costs associated with component replacement and maintenance can be significant [2,3].

The tribological performance of engineering materials is influenced by a combination of material properties, contact conditions, environmental factors, and operating parameters. During sliding contact, different wear mechanisms can occur, such as adhesive wear, abrasive wear, oxidative wear, and delamination wear [4,5]. These mechanisms may operate individually or simultaneously, depending on factors such as the applied load, sliding speed, sliding distance, surface roughness, and lubrication conditions. Therefore, understanding and accurately predicting wear behavior is essential for enhancing component reliability and optimizing engineering designs [6].

Low-carbon alloy steels are extensively used in mechanical and automotive components due to their strong balance of strength, toughness, ductility, and cost-effectiveness [7]. Common examples include EN3, 20MnCr5, and EN353, which are found in gears, shafts, and transmission elements that face severe loading and sliding contact [8]. These steels are ideal for carburizing and case hardening, resulting in hard, wear-resistant surfaces while keeping a tough core. Their widespread application highlights the importance of studying their tribological behavior for researchers and design engineers [9].

The experimental evaluation of wear characteristics is commonly conducted using a pin-on-disk tribometer following ASTM G99 standards [10]. This method enables systematic variation in parameters such as normal load, sliding velocity, and counterface material, allowing researchers to study their effects on wear rate and friction. While pin-on-disk tests provide reliable results, conducting numerous experiments across various parameter combinations can be time-consuming and costly, particularly in optimization studies [11].

To reduce experimental effort while maintaining prediction accuracy, researchers are increasingly turning to computational modeling and AI techniques for tribological analysis. Statistical methods, such as regression analysis and design of experiments, are effective in modeling wear behavior under specific conditions. However, these methods often struggle to accurately capture nonlinear relationships among interacting variables and tend to have limited predictive abilities when extrapolating beyond the experimental domain [12,13]. Recent studies demonstrate a strong shift toward integrating physics-informed machine learning and explainable AI to optimize energy efficiency, trajectory planning, and predictive wear analysis in industrial manipulators and tribological components [14]. Recent advances in machine learning have significantly enhanced predictive models for complex engineering systems. Techniques such as Artificial Neural Networks (ANNs), Support Vector Machines (SVMs), Random Forests (RFs), Gradient Boosting, and Deep Neural Networks (DNNs) have shown great success in predicting wear rates, friction coefficients, and material performance [15]. These models can learn nonlinear relationships without needing explicit mathematical formulations. However, they often require large amounts of high-quality data and may struggle with generalization under limited training data or new operating conditions [16].

Physics-Informed Neural Networks (PINNs) are an advanced machine learning framework that addresses the limitations of purely data-driven methods [17]. Unlike traditional neural networks, PINNs incorporate physical laws into the training process by embedding differential equations and constraints into the loss function. This approach helps the model align with both experimental data and the underlying physics, resulting in improved accuracy, faster convergence, and greater robustness, even with limited data [18,19].

Physics-Informed Neural Networks (PINNs) have demonstrated success in fields such as fluid dynamics, heat transfer, and structural mechanics, but their application in tribological studies remains limited. There have been few investigations utilizing PINNs to predict wear behavior in low-carbon engineering steels under varying conditions, revealing a significant gap in research. By integrating physical knowledge with experimental data, it may be possible to enhance predictive capabilities and reduce the need for costly laboratory experiments [12,20,21].

In this study, the steels EN3, 20MnCr5, and EN353 were machined into ASTM G99 pin specimens and tested under various loads, sliding speeds, and sliding distances using a pin-on-disk tribometer. The experimental wear data collected were used to develop a PINN model for wear prediction. The model’s accuracy was evaluated by comparing predicted wear values with experimental results to demonstrate the effectiveness of PINNs as a computationally efficient tool for intelligent tribological design in engineering materials [4,12].

Fretting slip and tribo-chemical degradation dominate surface tool failure under continuous extreme cutting conditions [3]. Integrating these contact physical insights into explainable, statistically validated tree-based machine learning models enables accurate data-driven wear prediction for carburized and non-carburized engineering steels. Future tribological systems are expected to incorporate digital twins, autonomous laboratories, multi-source data fusion, and adaptive learning algorithms [5]. These advancements will enable predictive models to be continuously updated with new data. As a result, intelligent wear prediction systems will be improved, supporting sustainable manufacturing, reducing maintenance costs, enhancing equipment reliability, and minimizing unplanned downtime.

The literature review highlights a significant gap in comprehensive experiments and data-driven analyses regarding the dry sliding wear behavior of carburized low-carbon alloy steels. Specifically, there has been a lack of comparison concerning the wear characteristics of EN3, 20MnCr5, and EN353 steels under identical conditions. Furthermore, the predictive capabilities of Decision Tree and Random Forest models have not been systematically assessed. Addressing these gaps is essential for developing reliable predictive tools for industrial applications.

Accordingly, the present investigation develops optimized Decision Tree and Random Forest regression models for predicting the dry sliding wear behavior of EN3, 20MnCr5, and EN353 steels in both as-received and carburized conditions. Experimental wear data were generated through ASTM G99 pin-on-disk tests conducted under different combinations of sliding speed, applied load, and test duration using a full factorial experimental design. Hyperparameter optimization was performed using GridSearchCV together with five-fold cross-validation to improve model generalization. The developed models were evaluated using Root Mean Square Error (RMSE), Mean Absolute Error (MAE), and the coefficient of determination (R^2^), while their robustness was further assessed through sensitivity analysis, confidence interval estimation, residual analysis, and feature importance evaluation. The proposed methodology combines experimental tribology with interpretable machine learning to establish an accurate, robust, and computationally efficient framework for predictive wear modeling of engineering steels. The findings of this study are expected to contribute to intelligent material selection, surface engineering, predictive maintenance, and data-driven manufacturing systems [22].

Surface quality inspection using ESN-YOLOv8 alongside tailored amorphous CrN/CrAlN multilayer coatings highlights how microstructural integrity and interface engineering mitigate surface degradation under severe wear and environmental stress [2]. Combining automated surface defect inspection with tailored multilayer coatings provides a comprehensive data foundation for training explainable tree-based machine learning models to accurately predict wear behavior across carburized and non-carburized engineering steels.

This study examines the wear behavior of EN3, 20MnCr5, and EN353 steels in both their as-received and carburized states under various conditions. Experimental data are utilized to create optimized models using Decision Tree and Random Forest algorithms. This involves hyperparameter tuning through GridSearchCV and five-fold cross-validation to enhance accuracy and robustness. The performance of the models is validated using techniques such as sensitivity analysis and confidence interval estimation. Additionally, a feature importance analysis identifies the key process parameters that influence wear behavior. This integrated approach combines experimental tribological research with interpretable machine learning, offering a reliable framework for predicting wear and assessing material performance. Recent machine learning advances in tribology predominantly rely on data-driven, tree-based models and explainable AI to predict complex wear rates and surface degradation in treated engineering steels under static operational parameters. In contrast, the proposed framework directly integrates differential physical laws and boundary constraints via Physics-Informed Neural Networks (PINNs), shifting the paradigm from purely empirical wear estimation to governing exact multi-objective system physics without relying solely on large experimental datasets.

## 2. Materials and Methods

### 2.1. Materials and Experimental Procedure

EN3, 20MnCr5, and EN353 steels were selected as the materials for the present investigation. The materials were supplied as hot-rolled round bars of 20 mm diameter in the annealed condition by Kanaka Alloys, Bangalore, Karnataka, India 560001. According to the manufacturer’s specifications, these steels are classified as low-carbon alloy structural steels and are widely employed in engineering applications because of their favorable combination of strength, toughness, machinability, and wear resistance. In addition, their excellent carburizing response makes them suitable for components subjected to severe contact and sliding conditions. The chemical compositions of the as-cast steel samples are presented in Table 1.

Wear test specimens were machined from the as-received bars using a CNC turning center (ACE Designers Limited, Peenya Industrial Area Bengalore) in accordance with ASTM G99 standards [23]. Cylindrical pin specimens having dimensions of 8 mm diameter and 35 mm length were prepared for dry sliding wear tests. Once the machining was done, the specimen surfaces were successively ground and polished using silicon carbide abrasive papers of increasing grit size to obtain a uniform surface finish. The specimens were then cleaned with acetone, supplied by Sri Durga Laboratory Equipment, Mangalore, Karnataka, 575006 to remove any surface contaminants before testing.

Dry sliding wear experiments were conducted using a pin-on-disk tribotester (Ducom TR-20 Neo Series) under ambient laboratory conditions. The wear behavior of both the as-received and carburized specimens of the three steel grades was evaluated to investigate the influence of carburizing on wear performance. Since 27 experiments were conducted for each test condition, only one test was conducted. During the tests, the pin specimens slid against a chromium carbide-coated steel disk with a hardness of approximately 74 HRC. A full factorial experimental design was employed to investigate the effects of the operating parameters on wear behavior. The selected process variables were sliding speed, applied load, and test duration, each considered at three levels. Sliding speeds of 200 rpm, 400 rpm, and 600 rpm, applied loads of 1.5 kg, 3.0 kg, and 4.5 kg, and test durations of 10 min, 20 min, and 30 min were used, resulting in a total of 27 experimental combinations. The wear response was evaluated under each experimental condition to determine the influence of the selected parameters and to compare the wear characteristics of the three steels in both the as-received and carburized conditions. The wear loss was measured by the weight-loss method using a digital electronic weighing machine, which has an accuracy of up to 4 decimal places. The non-carburized and carburized specimens used for wear tests are shown in Figure 1.

Wear tests were conducted in accordance with ASTM G-99 under laboratory conditions, with relative humidity maintained between 80% and 85% and temperature ranging from 25 °C to 28 °C. The disk material is a chromium carbide disk with a hardness of 74 HRC. The pin-and-disk machine is illustrated in Figure 2.

The carburizing process was conducted with parameters such as temperature, time and carbon potential. The furnace was stabilized at 930 °C for 30 min under a propane flow rate of 1.5 L/h. The specimens were then carburized in three stages: boosting at 930 °C for 12 h with 0.9–1.1% carbon potential, diffusion at 930 °C for 12 h with 0.8–0.9% carbon potential, and equalizing at 820 °C for 1 h–2 h with 0.6–0.7% carbon potential. After treatment, the specimens were furnace cooled to room temperature. The average hardness values of five readings for each specimen in the as-received condition are presented in Table 2. Once the carburization is done, the hardness is measured from core to case at a distance of 1 mm, and the values are presented in Table 3.

### 2.2. Development of Wear Prediction Model

The complete workflow adopted for developing and validating the data-driven wear prediction models using optimized tree-based machine learning techniques is illustrated in Figure 3. The methodology consists of sequential steps, starting from data acquisition to final result interpretation.

Table 4 presents the experimental wear data for various steel materials tested under different operating conditions, such as speed, load, and time. The dataset includes both as-received and carburized conditions, allowing for a comparison of wear performance across different material treatments. It is observed that mass loss generally increases with higher load and longer sliding time, while carburized samples demonstrate significantly lower wear due to their enhanced surface hardness. This dataset serves as the foundation for training and validating machine learning models for wear prediction.

### 2.3. Dataset and Preprocessing

The dataset consists of experimentally obtained wear values under varying operating conditions, including load, time, speed, and material condition. Feature engineering included the creation of interaction terms such as load × time to capture combined effects.

### 2.4. Model Development

Two tree-based machine learning models, namely the Decision Tree (DT) and Random Forest (RF), were developed to predict wear behavior in steel materials. To enhance model performance and ensure optimal generalization, hyperparameter tuning was carried out using GridSearchCV in conjunction with 5-fold cross-validation. This approach systematically evaluates multiple combinations of hyperparameters and selects the configuration that yields the best cross-validation performance. For the Decision Tree model, the optimal parameters were identified as a maximum depth of 5, a minimum of 2 samples required to split an internal node, and a minimum of 1 sample at each leaf node. In the case of the Random Forest model, the optimal configuration consisted of 300 estimators (trees), no restriction on maximum depth, a minimum of 2 samples required for node splitting, and a minimum of 1 sample per leaf. These optimized parameters ensure a balance between model complexity and generalization capability, thereby improving predictive accuracy while minimizing the risk of overfitting.

### 2.5. Evaluation Metrics

Model performance was evaluated using three widely accepted statistical metrics: Root Mean Square Error (RMSE), Mean Absolute Error (MAE), and the coefficient of determination (R^2^). RMSE calculates the square root of the average of the squared differences between predicted and actual values, giving more weight to larger errors. MAE measures the average absolute difference between predicted and observed values, offering a straightforward assessment of prediction accuracy. The R^2^ score indicates the proportion of variance in the dependent variable explained by the model, reflecting its overall goodness of fit. Together, these metrics provide a comprehensive evaluation of a model’s accuracy, robustness, and predictive capability.

## 3. Results and Discussion

The custom physics-informed loss function enforces an energy/work interaction penalty where normalized mass loss prediction (yˆ) is soft-constrained to align with the normalized product of applied load (F) and time (t), reflecting Archard’s wear principles:

$$L_{\mathrm{total}}=\frac{1}{N}\sum_{i=1}^{N}{\left(y_{i}-{\hat{y}}_{i}\right)}^{2}+0.1\cdot \frac{1}{N}\sum_{i=1}^{N}{\left(\frac{{\hat{y}}_{i}}{\max\left(\hat{y}\right)+\epsilon }-\frac{F_{i}\cdot t_{i}}{\max\left(F\cdot t\right)+\epsilon }\right)}^{2}$$

To evaluate the predictive capability of different machine learning approaches for wear estimation, Decision Tree (DT), Random Forest (RF), and Physics-Informed Neural Network (PINN) models were developed and compared using statistical performance metrics and explainable artificial intelligence (XAI) techniques. The models were assessed based on their prediction accuracy, feature interpretability, and robustness using RMSE, MAE, R^2^, SHAP analysis, and graphical comparisons.

The comparative results provide insights into the influence of process parameters on wear prediction and identify the most suitable algorithm for the developed experimental dataset. The following subsections discuss the interpretation of each figure in detail.

The wear values that were measured experimentally and the predictions made by the Decision Tree, Random Forest, and PINN models are contrasted in Figure 4. The perfect prediction, when the projected values precisely match the experimental observations, is shown by the dashed diagonal line. Excellent prediction accuracy is demonstrated by the Random Forest forecasts’ narrow distribution around the ideal line. Despite a slightly greater divergence, the Decision Tree model likewise shows good agreement with the experimental findings. The PINN forecasts, on the other hand, show significant dispersion from the ideal line, indicating poorer generalization performance and prediction accuracy. The discrete distribution of the PINN prediction results in Figure 4 is attributed to the nonlinear and coupled effects of the input parameters on wear behavior and does not indicate random model predictions. During training, the PINN loss was continuously monitored and showed a stable decreasing trend, followed by convergence to a nearly constant value. This confirms that the model achieved a stable solution during the training process. The observed variation in the predicted values is therefore mainly associated with the complex relationship between the input parameters and the wear response.

The Root Mean Square Error (RMSE) from the three predictive models is contrasted in Figure 5. The Random Forest model outperformed the other algorithms in terms of prediction accuracy and generalization capacity, as evidenced by its lowest RMSE. With only a minor decline in performance, the Decision Tree model likewise yielded quite a low prediction error. The Physics-Informed Neural Network (PINN), on the other hand, showed a significantly higher RMSE, suggesting that the projected and experimentally measured wear values did not correspond well. The increased prediction error implies that the wear characteristics from the comparatively limited experimental dataset were not successfully learned by the installed PINN.

Figure 6 presents the residual plots of the Decision Tree, Random Forest, and PINN models using the independent test dataset. The Decision Tree and Random Forest residuals are generally distributed around the zero-residual line, indicating comparatively satisfactory prediction performance, although a few larger deviations are observed. In contrast, the PINN residuals exhibit a clear systematic decreasing trend with increasing predicted wear, rather than a random distribution. This behavior indicates that the current PINN formulation does not fully capture the nonlinear relationship between the input parameters and wear response, particularly at higher and lower wear values. Therefore, the residual analysis provides additional evidence of the differences in predictive behavior among the three models beyond the RMSE, MAE, and R^2^ metrics.

Using the evaluation measures R^2^, RMSE, and MAE, Figure 7 summarizes the three models’ overall predictive performance. By obtaining the best coefficient of determination (R^2^) and the lowest prediction errors (RMSE and MAE), the Random Forest model has the most balanced performance. With only a slight drop in prediction accuracy, the Decision Tree model likewise exhibits competitive performance. The PINN model, on the other hand, performs much worse due to its higher error metrics and lower R^2^ value. The radar chart unequivocally demonstrates the Random Forest algorithm’s advantage for wear prediction in the current testing setup.

The global feature importance derived from the optimized Random Forest model and the SHAP (SHapley Additive exPlanations) summary plot are shown in Figure 8. The input factors are ranked by their impact on the expected wear values in the SHAP summary plot (top panel). The color scale shows the magnitude of the feature values, with red indicating higher values and blue indicating lower values. Each point represents a single observation. The anticipated wear is higher for features with positive SHAP values and lower for those with negative SHAP values. Carburization modifies several material characteristics simultaneously, particularly surface hardness, case depth, microstructure, and surface mechanical properties. Therefore, the high SHAP value associated with the carburization-related feature reflects the combined effect of these changes on wear resistance rather than the influence of a single physical property.

The average contribution of each characteristic to the model predictions is further quantified in the global SHAP significance plot (bottom panel). The most important factors influencing wear behavior are found to be condition (carburized) and material (L27 EN3 steel), followed by time (min) and the designed interaction feature load × time. By contrast, the contributions of load () and speed (RPM) are comparatively lower. These findings support the Random Forest model’s ability to capture intricate nonlinear interactions between the experimental variables by showing that both material properties and operating conditions primarily govern the wear response.

The SHAP dependence plot, which shows how applied load affects the expected wear values, is shown in Figure 9. A single experimental observation is represented by each point, and the appropriate material type is indicated by the color. The distribution of SHAP values shows that the applied load has a nonlinear effect that varies according to the type of material. The Random Forest model captures the interaction effects because, while increasing load generally leads to increased wear, the amount of this contribution varies depending on the material conditions. The use of ensemble learning techniques for the interpretation of tribological data is justified by such nonlinear behavior.

The comparative evaluation shows that both the Random Forest and Decision Tree models consistently outperform the Physics-Informed Neural Network (PINN) in predicting wear behavior. While PINNs excel in applications governed by well-defined physical equations and large datasets, the current experimental dataset is relatively small and lacks sufficient information for effective physics-constrained learning. As a result, the PINN model displayed significantly higher Root Mean Square Error (RMSE) and Mean Absolute Error (MAE) values, along with a lower coefficient of determination (R^2^), indicating poor predictive performance. Due to these limitations, only the Decision Tree and Random Forest models were selected for the subsequent sensitivity analysis and hyperparameter optimization. These models demonstrated superior prediction accuracy, robustness, and reliability, making them more suitable for detailed performance evaluation and optimization in the current wear prediction study.

### 3.1. Sensitivity to Data Splitting

The sensitivity of the developed models to data partitioning was assessed by varying the test size from 0.1 to 0.4. The results indicated that the coefficient of determination (R^2^) remained relatively stable across different split ratios, suggesting consistent predictive performance. The minimal variation in R^2^ values demonstrates that the models are not highly dependent on a specific train–test configuration. This low sensitivity to data splitting confirms the robustness and reliability of the proposed machine learning models for wear prediction.

Table 5 presents the results of a sensitivity analysis conducted by varying the test size from 0.1 to 0.4 for both the Decision Tree (DT) and Random Forest (RF) models. The performance of the models was evaluated using Root Mean Square Error (RMSE), Mean Absolute Error (MAE), and the coefficient of determination (R^2^). From the table, it is evident that at lower test sizes (0.1 and 0.2), both models demonstrate relatively low R^2^ values, particularly for the Random Forest model, which has R^2^ values of 0.2043 and 0.2428, respectively. These low values indicate limited generalization due to insufficient validation data. As the test size increases, the performance of both models improves significantly. At a test size of 0.3, the Decision Tree achieves an R^2^ of 0.6406, while the Random Forest outperforms it with an R^2^ of 0.7332. The best performance occurs at a test size of 0.4, where the Decision Tree reaches an R^2^ of 0.7114, and the Random Forest achieves the highest R^2^ of 0.8059.

Error metrics (RMSE and MAE) show a similar trend, with the Random Forest demonstrating relatively lower error values at higher test sizes, indicating better predictive accuracy and stability. Overall, the Random Forest model exhibits improved generalization capability compared to the Decision Tree, particularly as the test size increases.

The variation in R^2^ with respect to test size for both models is shown graphically in Figure 10. Improved model reliability with bigger validation sets is highlighted by the figure, which clearly demonstrates an upward trend in R^2^ as the test size grows. Furthermore, the Random Forest curve shows less susceptibility to data partitioning, appearing smoother and more consistent. The Decision Tree model, on the other hand, has somewhat larger variability, indicating a stronger reliance on the distribution of training data.

### 3.2. Effect of Hyperparameter Optimization

Hyperparameter tuning significantly improved performance:

- Optimal tree depth (DT) = 5 prevents overfitting;
- Increasing in estimators (RF) improves stability and is optimal at 300.

The impact of maximum tree depth on the Decision Tree model’s performance under various minimum sample split circumstances is shown in Figure 11. As the tree depth rises from 2 to 5, it is shown that the model performance (R^2^) considerably improves, suggesting improved learning of nonlinear interactions. Nevertheless, the improvement becomes negligible after a depth of 5, indicating the beginning of overfitting. Five is shown to be the ideal depth, offering a balance between variance and bias.

The impact of changing the number of trees (estimators) on the Random Forest model’s performance for various maximum depth values is displayed in Figure 12. The findings show that adding more estimators improves model performance and stability, especially up to 300 trees. Improvements are negligible after this, demonstrating that 300 estimators offer the best balance between computational cost and accuracy. Unrestricted depth models (max_depth = None) attain the highest.

### 3.3. Feature Importance Analysis

The Random Forest model’s feature significance ratings are shown in Figure 13. The interaction term that contributes most to wear prediction is load × time. While speed has a relatively small impact, load and time independently also play an important role. Wear regimes can also be distinguished by material-related characteristics. These results are in line with recent tribology research, which highlights the intricate and interconnected nature of tribological systems by demonstrating through machine learning models that wear behavior is controlled by several interacting parameters, including load, sliding distance, and operating circumstances [6].

The partial dependency of wear on the three main input variables, such as load, time, and speed, is shown in Figure 14. The findings show that wear rises as load and sliding time increase, although there is a nonlinear connection with speed. These patterns are in line with traditional tribological theory, especially Archard’s Wear Law [6], which asserts that wear is proportionate to sliding distance and applied load. Higher wear is anticipated as sliding distance increases over time. The nonlinear behavior with speed can be attributed to competing mechanisms such as frictional heating, oxidation, and variations in contact conditions at different velocities. These findings demonstrate that the model effectively captures the complex interactions governing wear behavior.

The correlation matrix between the target variable and the input features is displayed in Figure 15. Individual factors show moderate correlations, while wear and the load × time interaction show strong positive relationships. This is explained by the combined effect of sliding distance and load, which controls material removal more strongly than either factor alone. Low multicollinearity, which enhances model stability and prediction reliability, is indicated by the lack of strong inter-feature correlations. Such behavior is in keeping with current research in data-driven wear modeling [6], where interaction effects dominate and reduced multicollinearity enhances machine learning performance.

## 4. Statistical Validation and Uncertainty Analysis

The Random Forest model’s projected and actual wear values are compared in Figure 16. High prediction accuracy is indicated by the data points’ close alignment along the diagonal reference line. The model’s capacity to approximate the underlying functional link between input variables and wear is reflected in this behavior. Residual errors, which are anticipated in experimental datasets because of noise and measurement uncertainty, are represented by small departures from the ideal line. The lack of systematic divergence implies that the model is objective and performs well when applied to new data.

### 4.1. Confidence Interval Estimation

Figure 17 illustrates the prediction results along with the 95% confidence interval. The confidence interval is computed using:


CI = 1.96·σ/√n

The relatively narrow confidence band indicates low prediction uncertainty and high reliability of the model. The widening of the interval at higher wear values reflects increased variability in extreme conditions, which is consistent with tribological systems where wear mechanisms become more complex at higher loads and sliding distances.

### 4.2. Model Performance Evaluation

A 70:30 train–test split was employed to evaluate the performance of the developed models. The cross-validation results indicate that the Decision Tree model achieved an R^2^ value of 0.794, while the Random Forest model attained an R^2^ value of 0.792. These comparable values demonstrate that both models are capable of effectively capturing the underlying nonlinear relationships governing wear behavior, indicating strong predictive capability and generalization performance.

Table 6 summarizes the quantitative performance metrics (RMSE, MAE, and R^2^). The Random Forest model achieves lower error values (RMSE = 0.00495; MAE = 0.00273) and higher R^2^ (≈0.724), demonstrating superior generalization compared to the Decision Tree model. This improvement is theoretically attributed to the ensemble learning mechanism, which reduces variance by averaging multiple decision trees.

The Decision Tree and Random Forest models’ R^2^ values with 95% confidence intervals are displayed in Figure 18. While Random Forest’s slightly narrower interval shows lower variance as a result of ensemble averaging, the overlapping error bars show that both models have similar predictive power.

## 5. Conclusions

In this study, we developed Decision Tree (DT), Random Forest (RF), and Physics-Informed Neural Network (PINN) models to predict the wear behavior of a material under various operating conditions. We evaluated these models using statistical performance metrics, including the coefficient of determination (R^2^), Root Mean Square Error (RMSE), and Mean Absolute Error (MAE). Additionally, we conducted SHAP analysis to interpret the contributions of different features.

Among the algorithms investigated, the Random Forest model demonstrated the highest predictive accuracy, achieving the highest R^2^ value alongside the lowest RMSE and MAE. This ensemble learning approach effectively captured the nonlinear relationships between operating parameters and wear behavior, leading to robust and reliable predictions. The Decision Tree model also showed satisfactory predictive capability, although its performance was slightly lower than that of the Random Forest model due to its greater susceptibility to overfitting.

The Physics-Informed Neural Network (PINN) incorporated a physics-based constraint on the interaction between load and sliding time into its loss function. However, the PINN did not perform well with the current experimental dataset, exhibiting a significantly lower coefficient of determination and much higher RMSE and MAE than the Decision Tree and Random Forest models. This relatively poor performance may be attributed to the limited size of the experimental dataset and the simplified physics constraint used in this study. PINNs typically require larger datasets and more comprehensive governing physical equations to fully leverage their strengths in creating physics-consistent representations.

The SHAP analysis revealed that surface condition (carburized) and material type (L27 EN3 steel) were the most influential parameters affecting wear prediction. These were followed by sliding time and the engineered interaction feature (load × time). These findings indicate that both material characteristics and operating conditions significantly influence wear behavior and validate the interpretability of the Random Forest model.

For applications in material design and predictive maintenance, the suggested framework serves as a reliable, accurate, and comprehensible tool. Future studies could enhance model generality by expanding the dataset to include a broader range of materials and operating conditions. Furthermore, predictive capabilities and interpretability could be improved by incorporating advanced techniques such as deep learning, hybrid models, and physics-informed machine learning. The development of digital twin systems and the utilization of real-time sensor data may also facilitate continuous wear monitoring and better decision-making in industrial settings.

## Figures and Tables

**Figure 1:**
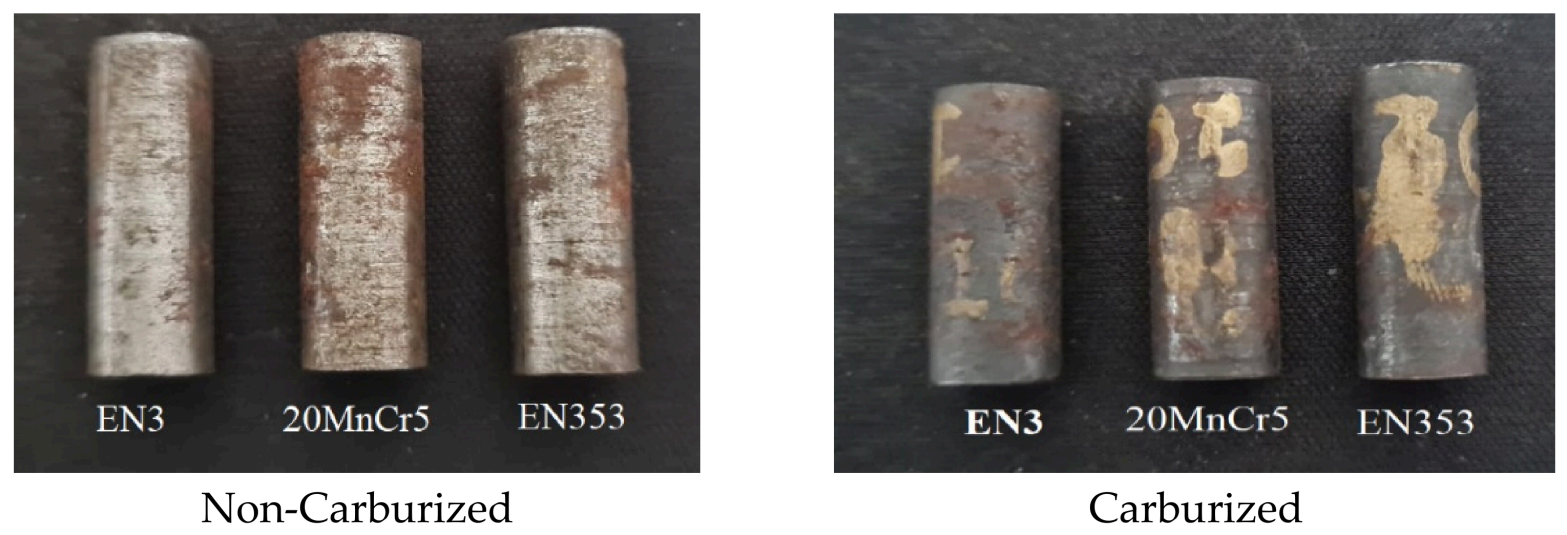
Non-carburized and carburized specimens used in the experimentation.

**Figure 2:**
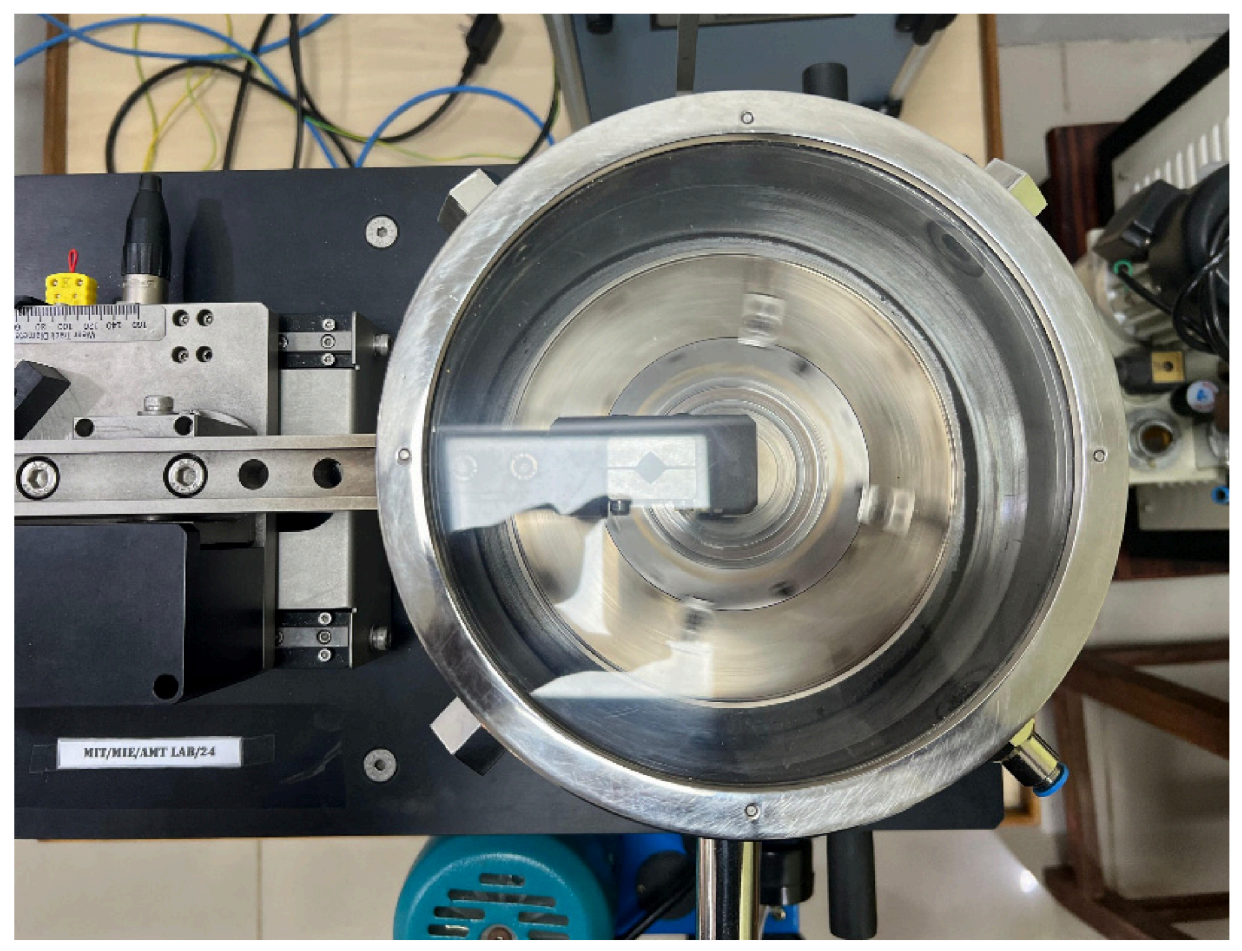
Pin-and-disk wear measuring instrument.

**Figure 3:**
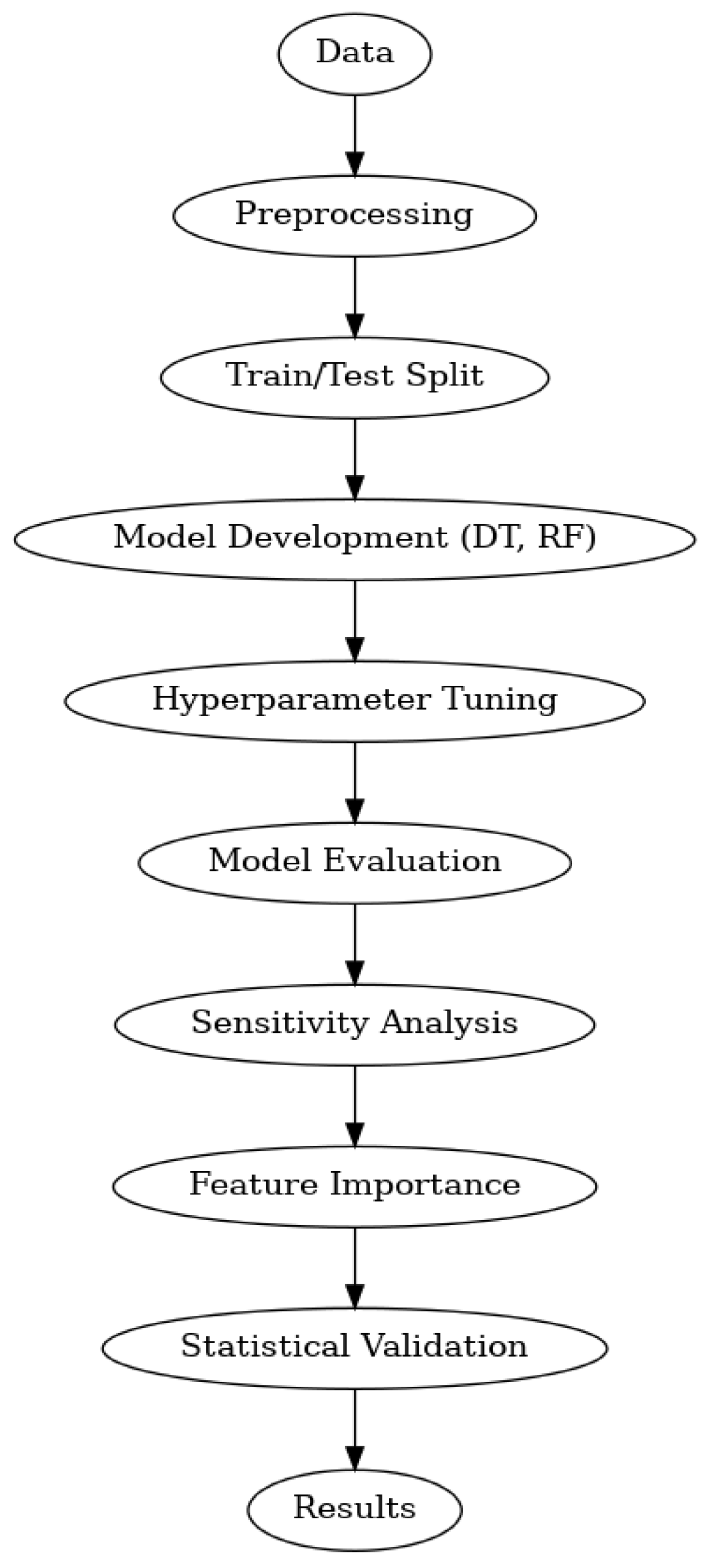
Wear model development flowchart.

**Figure 4:**
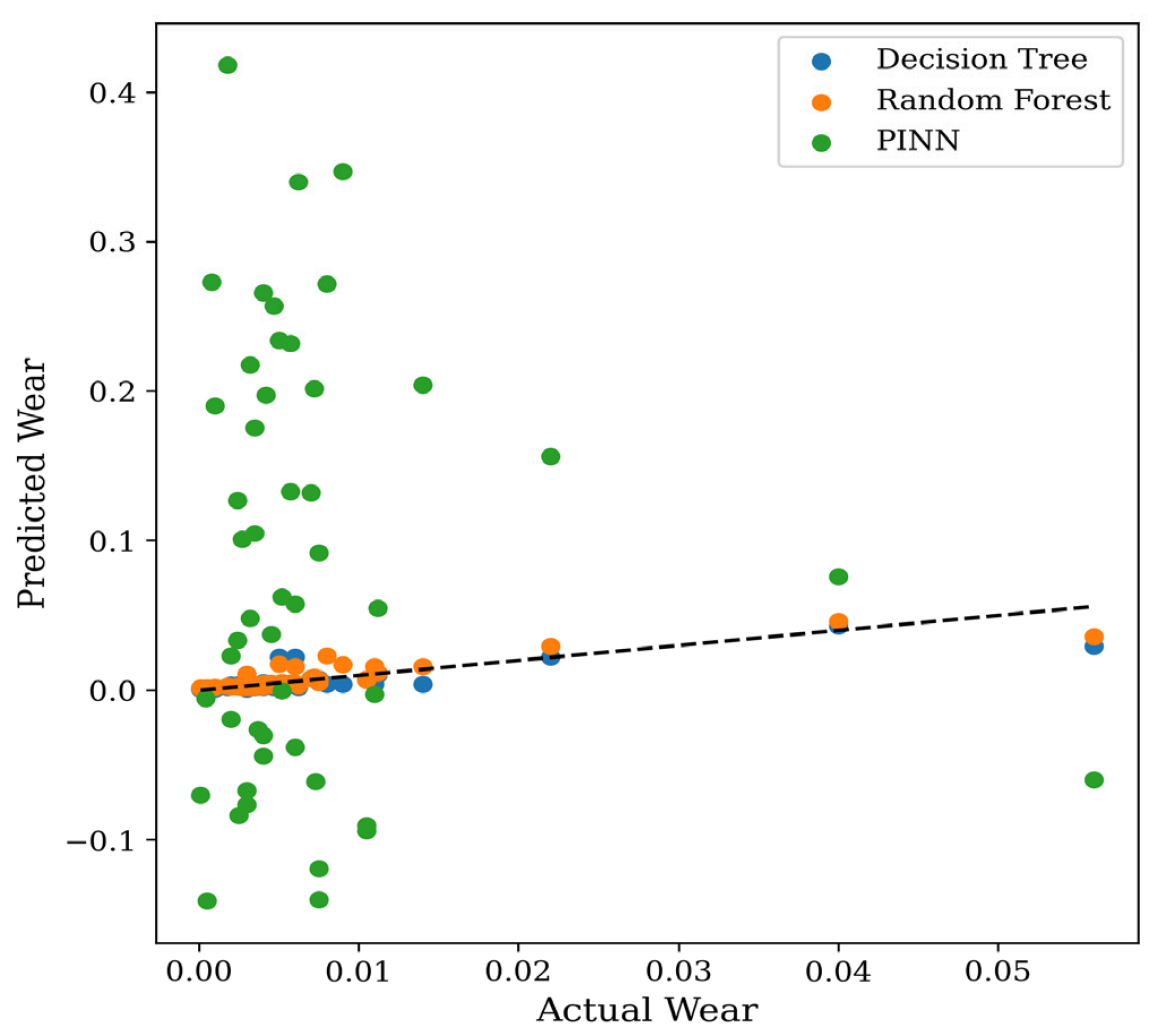
Comparison between actual and predicted wear values.

**Figure 5:**
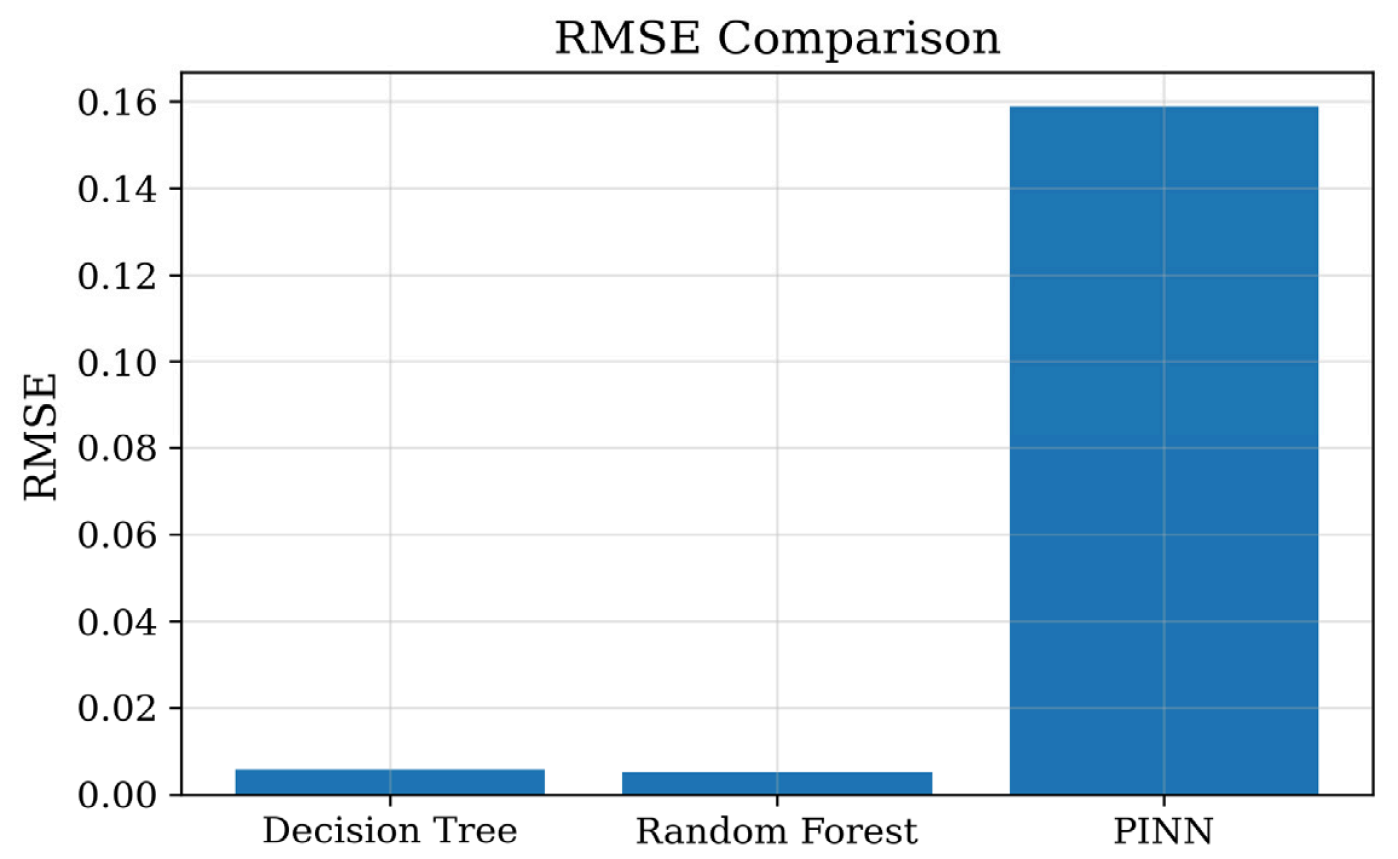
Comparison of RMSE for Decision Tree, Random Forest, and PINN models.

**Figure 6:**
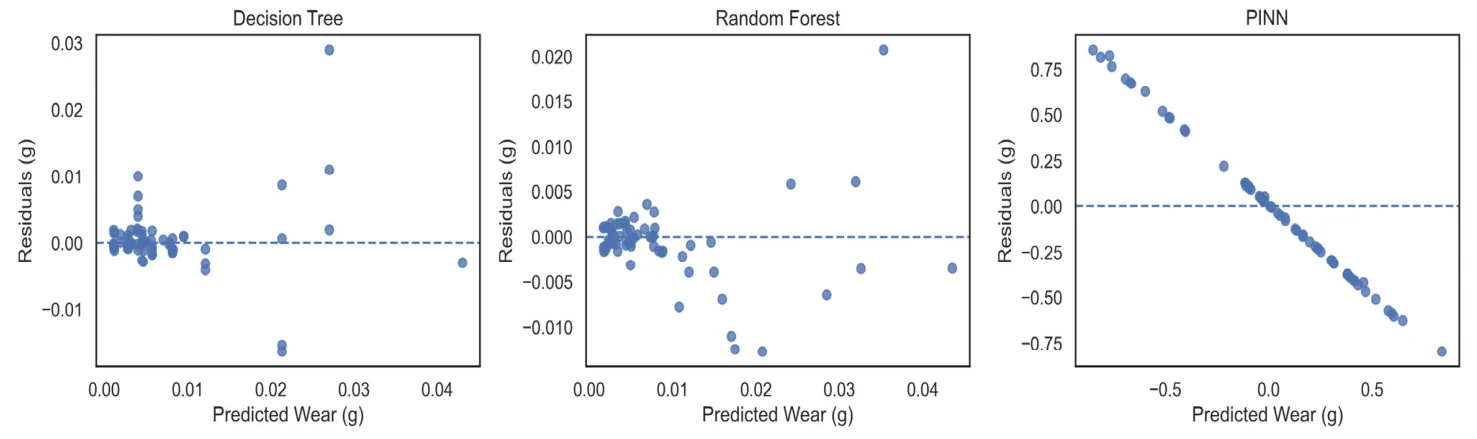
Residual analysis of Decision Tree, Random Forest, and PINN models.

**Figure 7:**
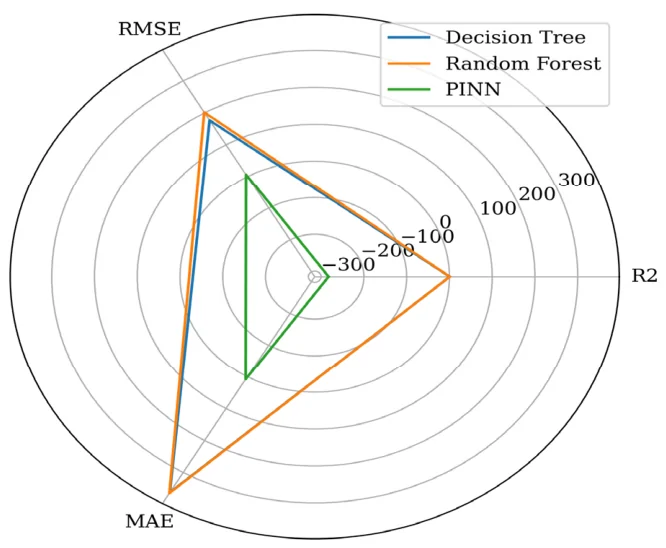
Radar chart comparing the performance of Decision Tree, Random Forest, and PINN models.

**Figure 8:**
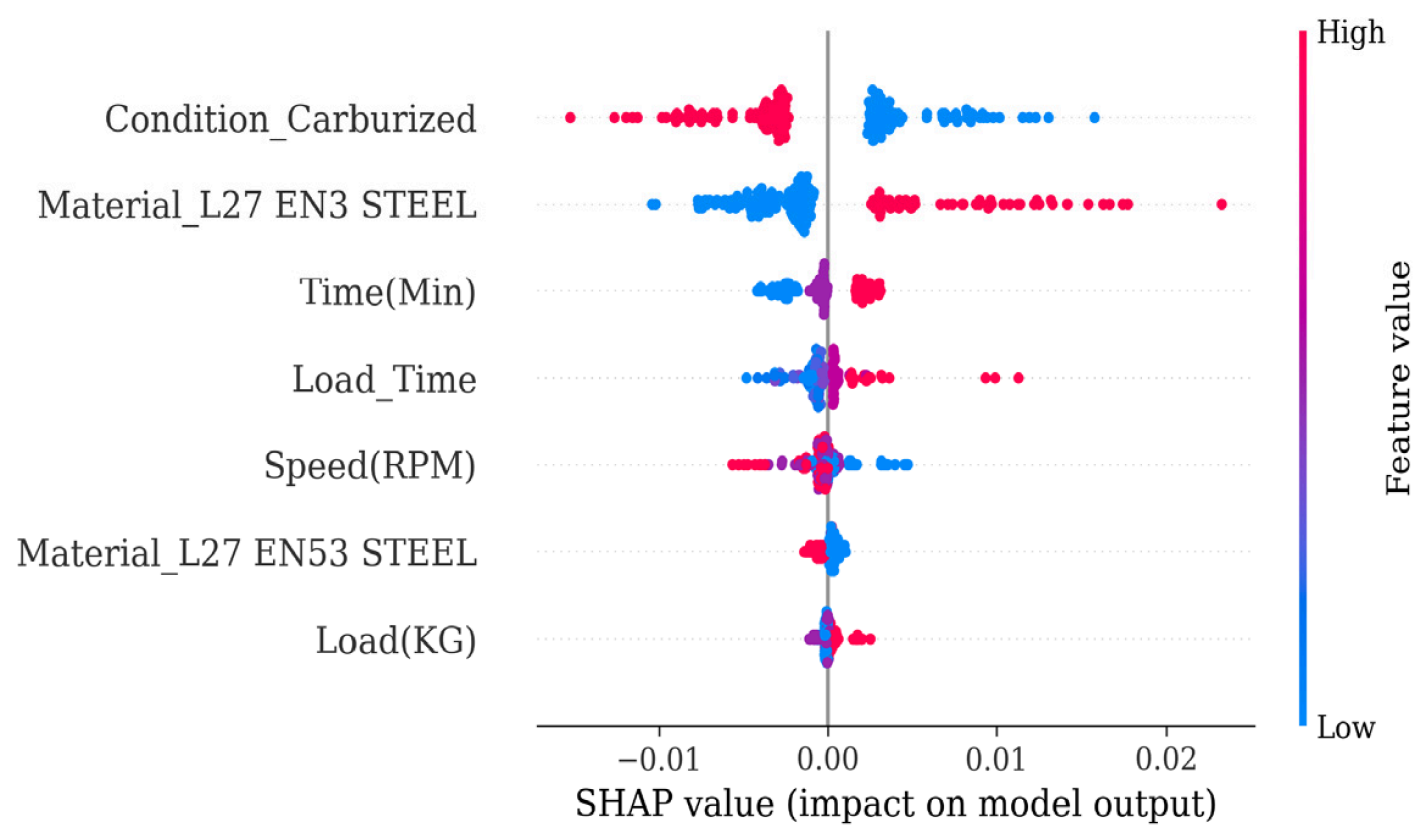
SHAP summary plot and global feature importance of the Random Forest model.

**Figure 9:**
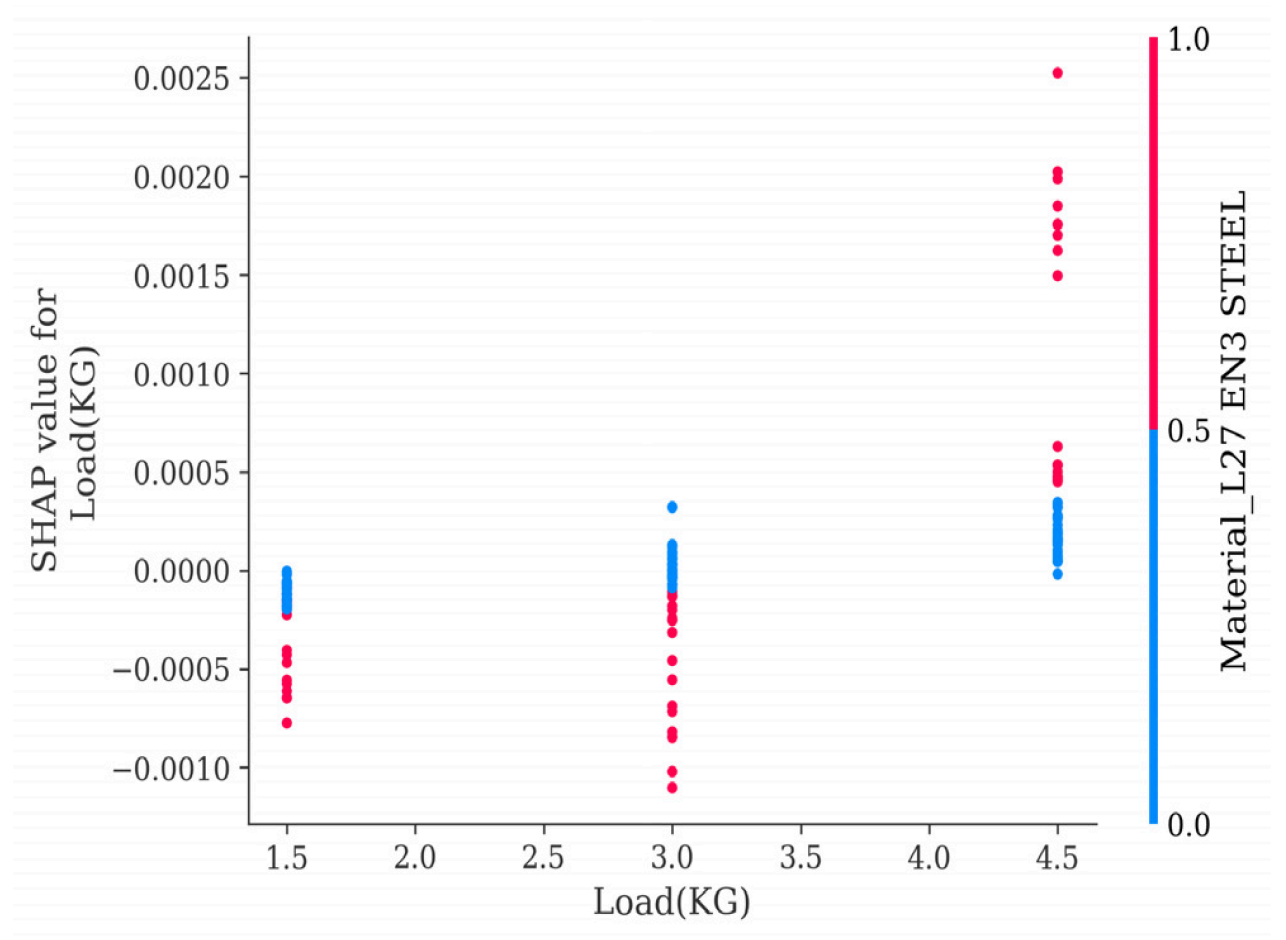
SHAP dependence plot for applied load.

**Figure 10:**
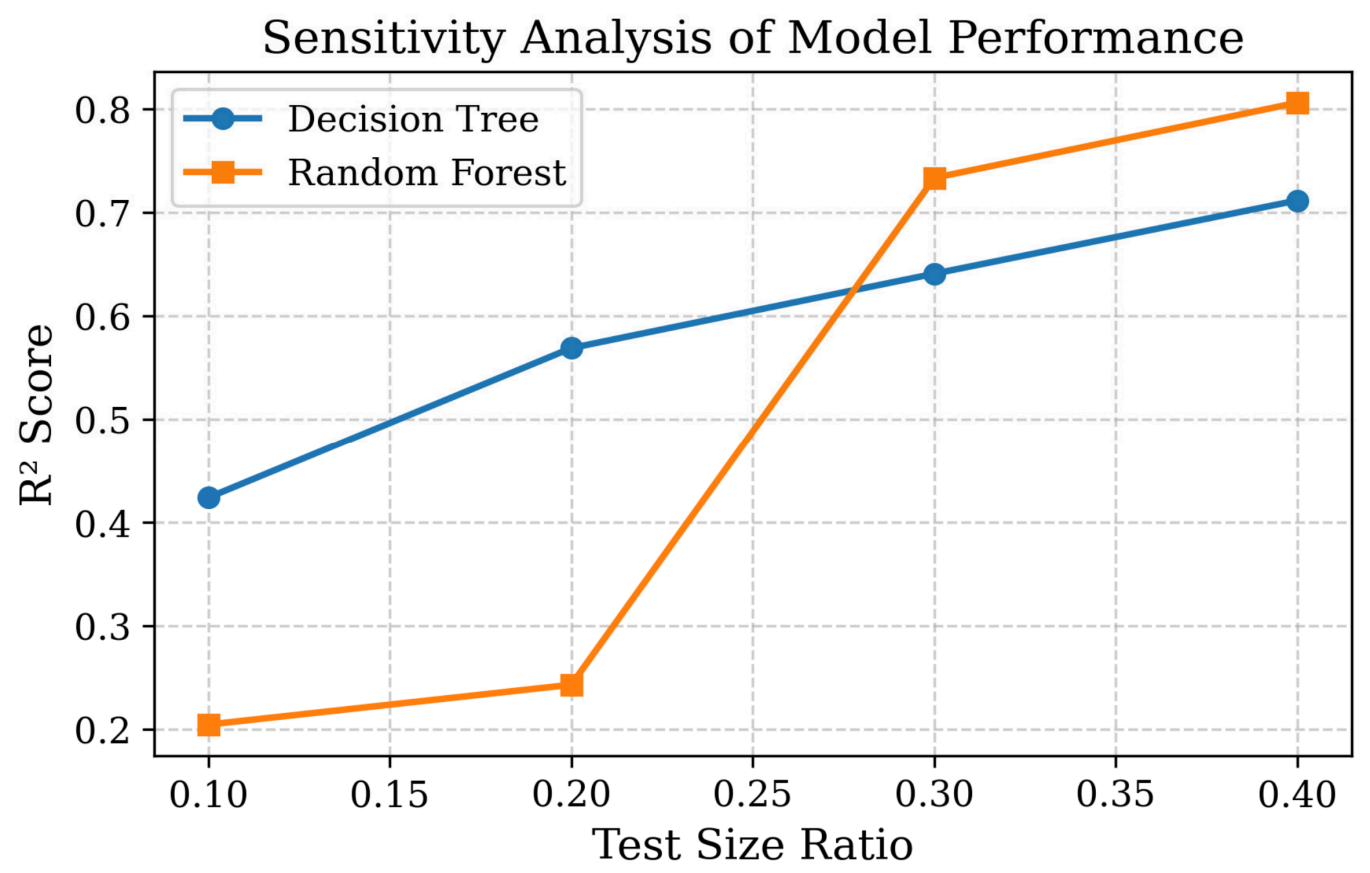
Sensitivity analysis showing variation in R^2^ with test size for Decision Tree and Random Forest.

**Figure 11:**
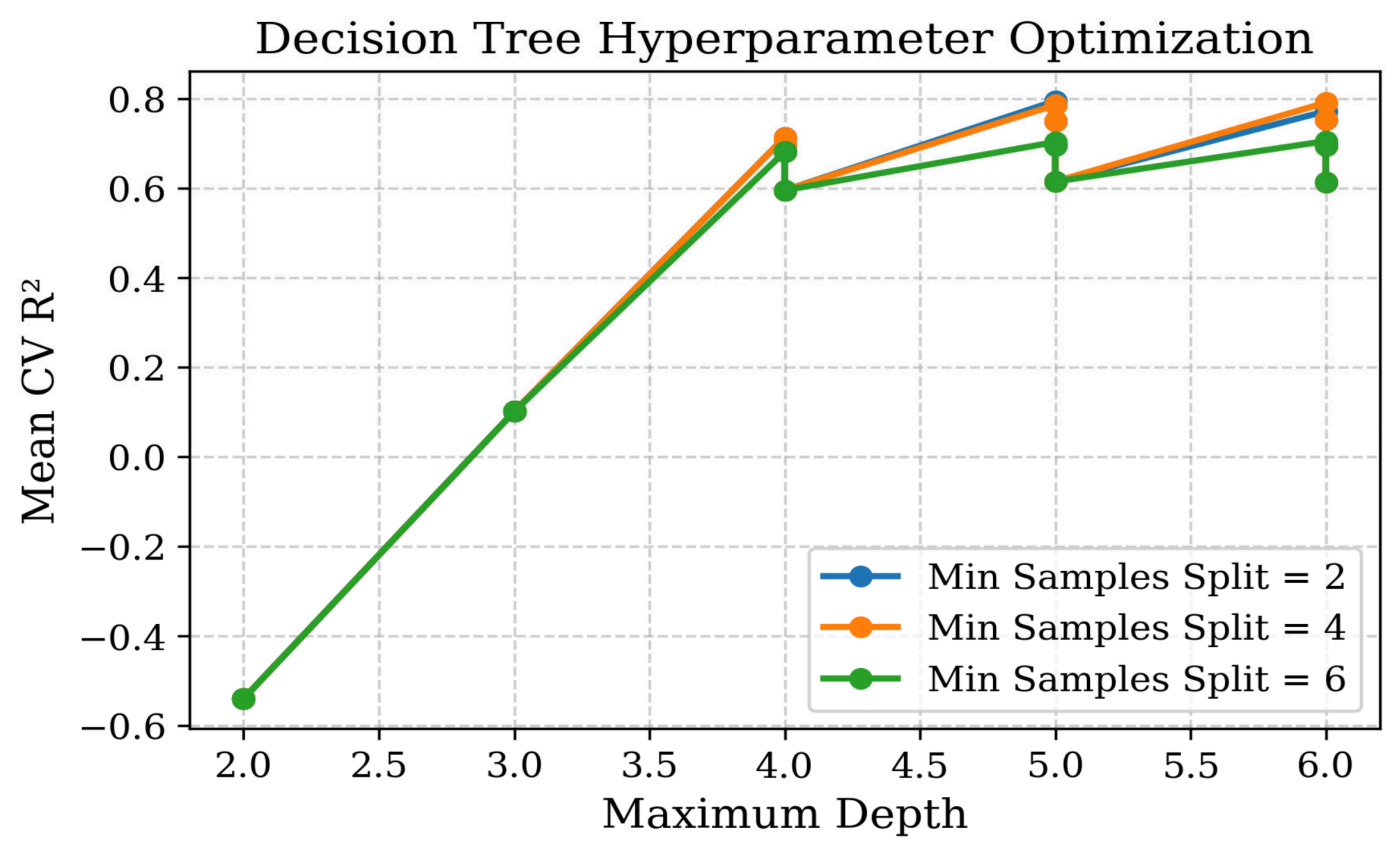
Effect of tree depth on Decision Tree.

**Figure 12:**
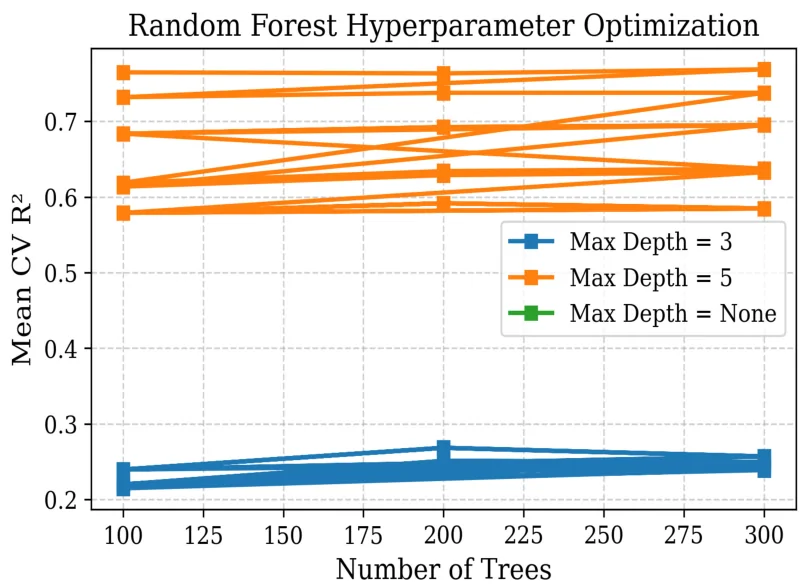
Influence of estimators on Random Forest.

**Figure 13:**
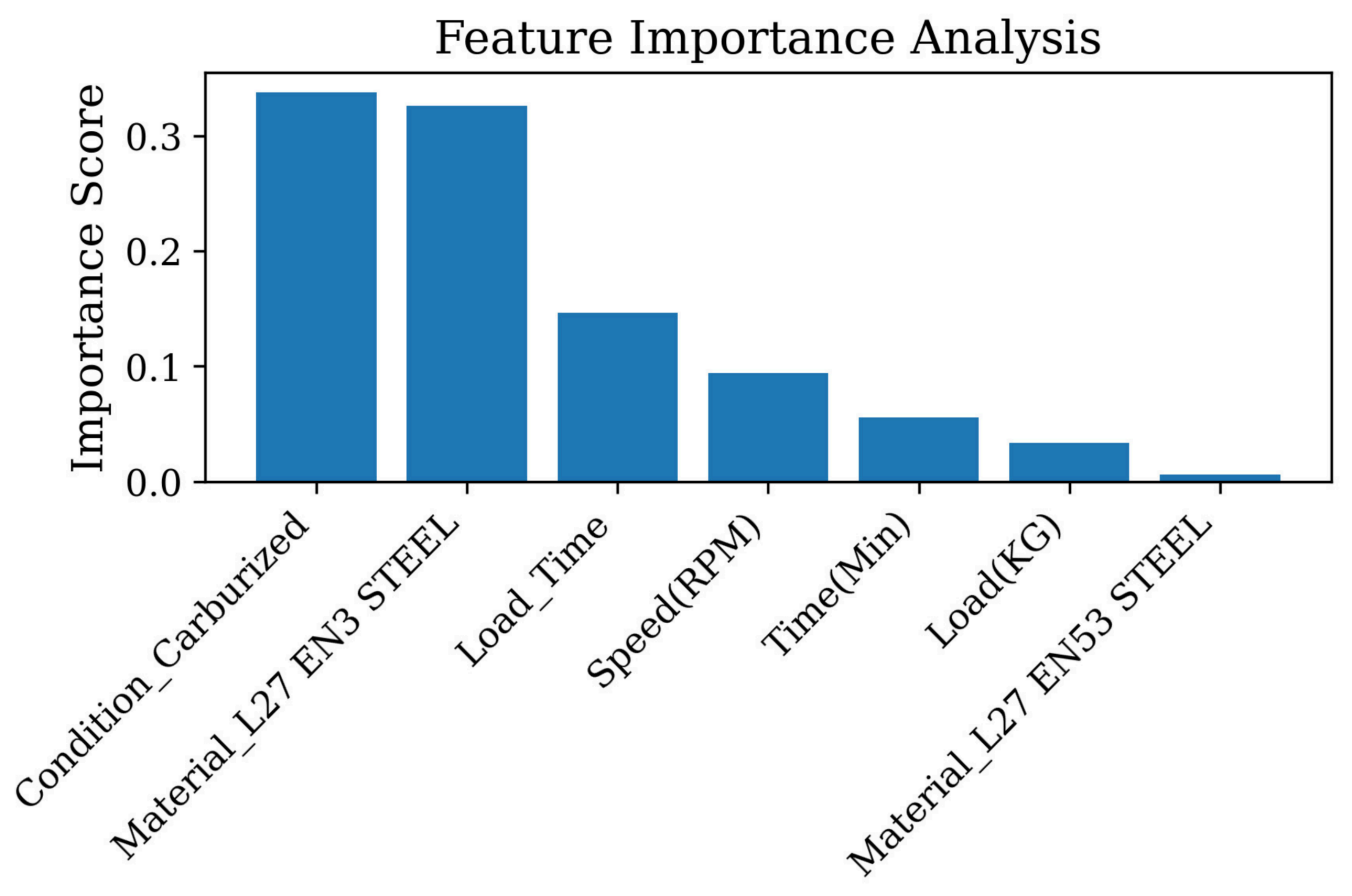
Feature importance plot.

**Figure 14:**
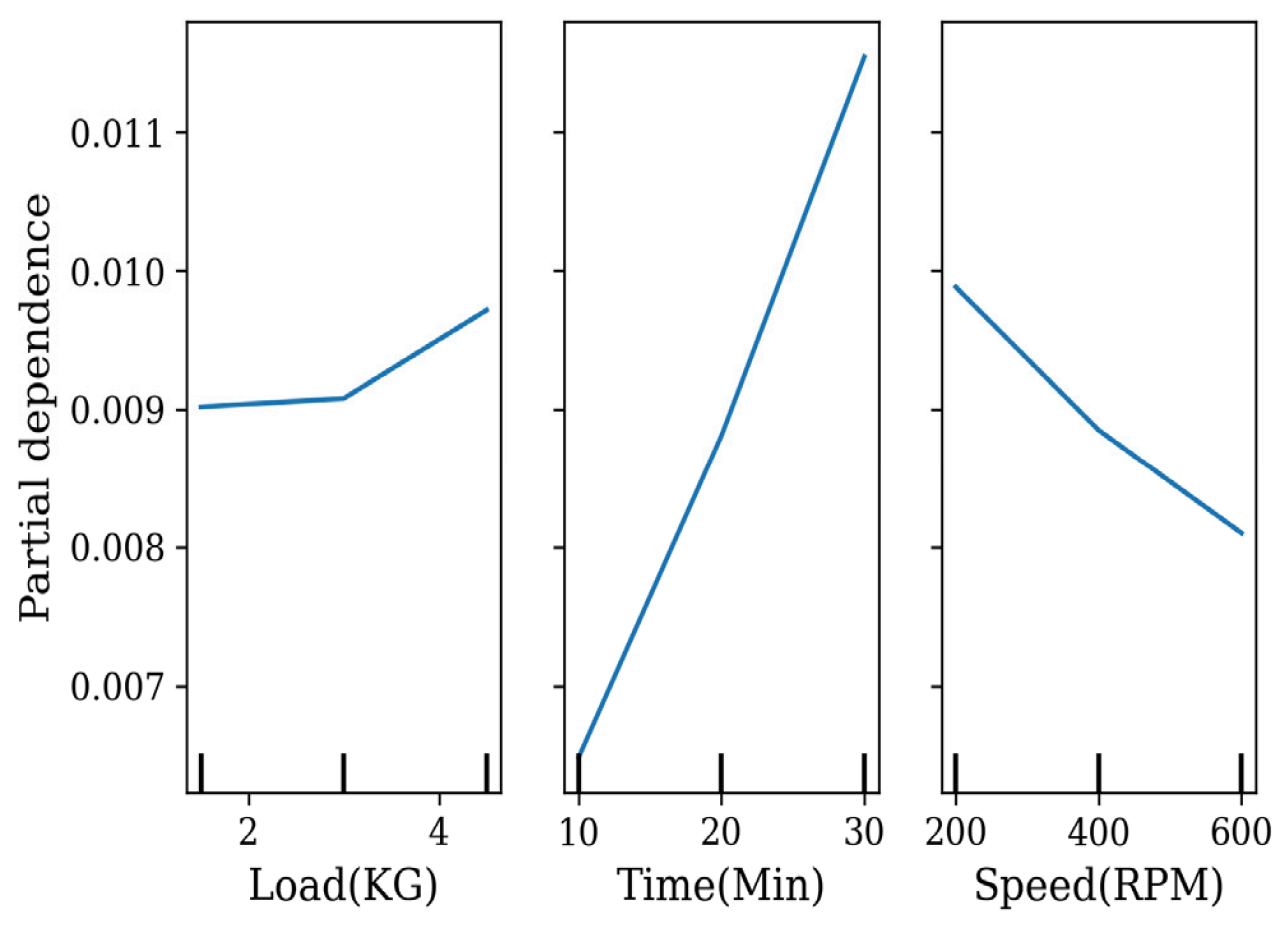
Dependence plot.

**Figure 15:**
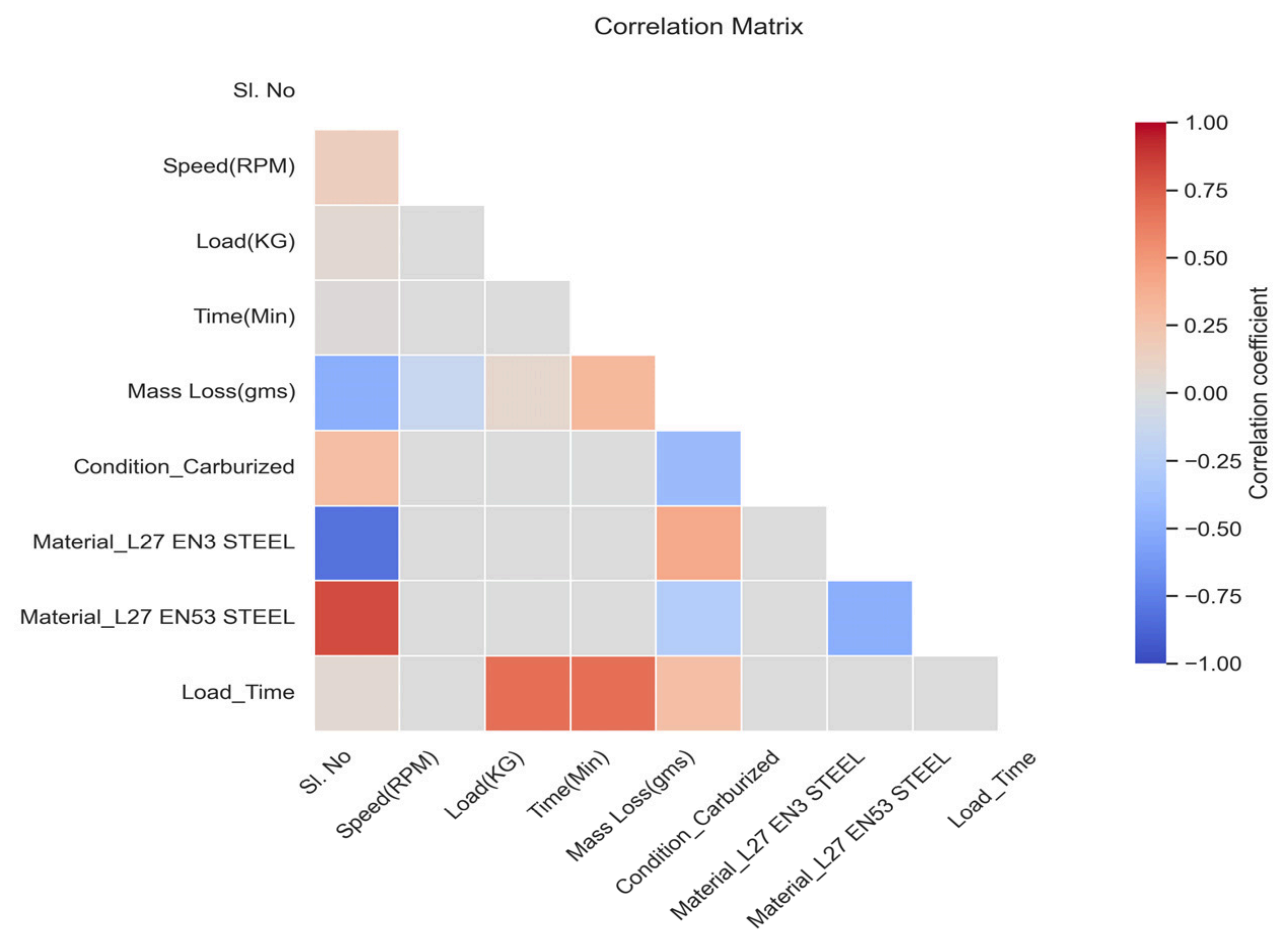
Correlation matrix.

**Figure 16:**
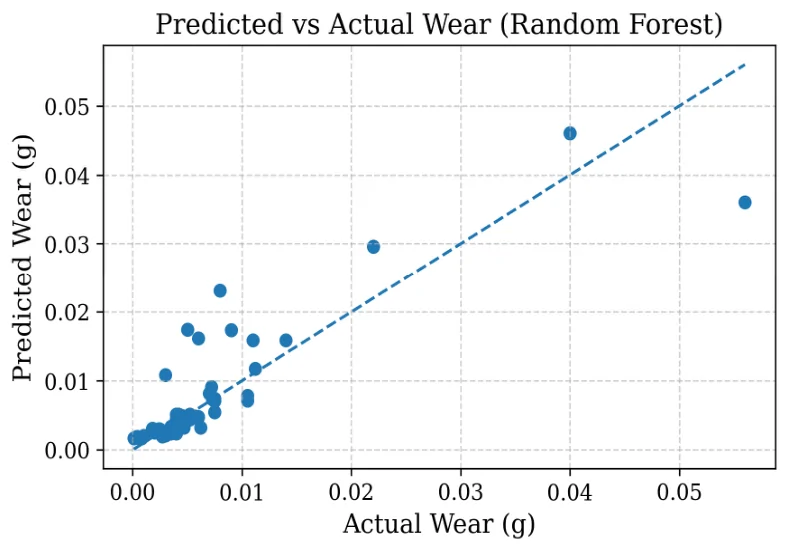
Predicted vs. actual wear comparison.

**Figure 17:**
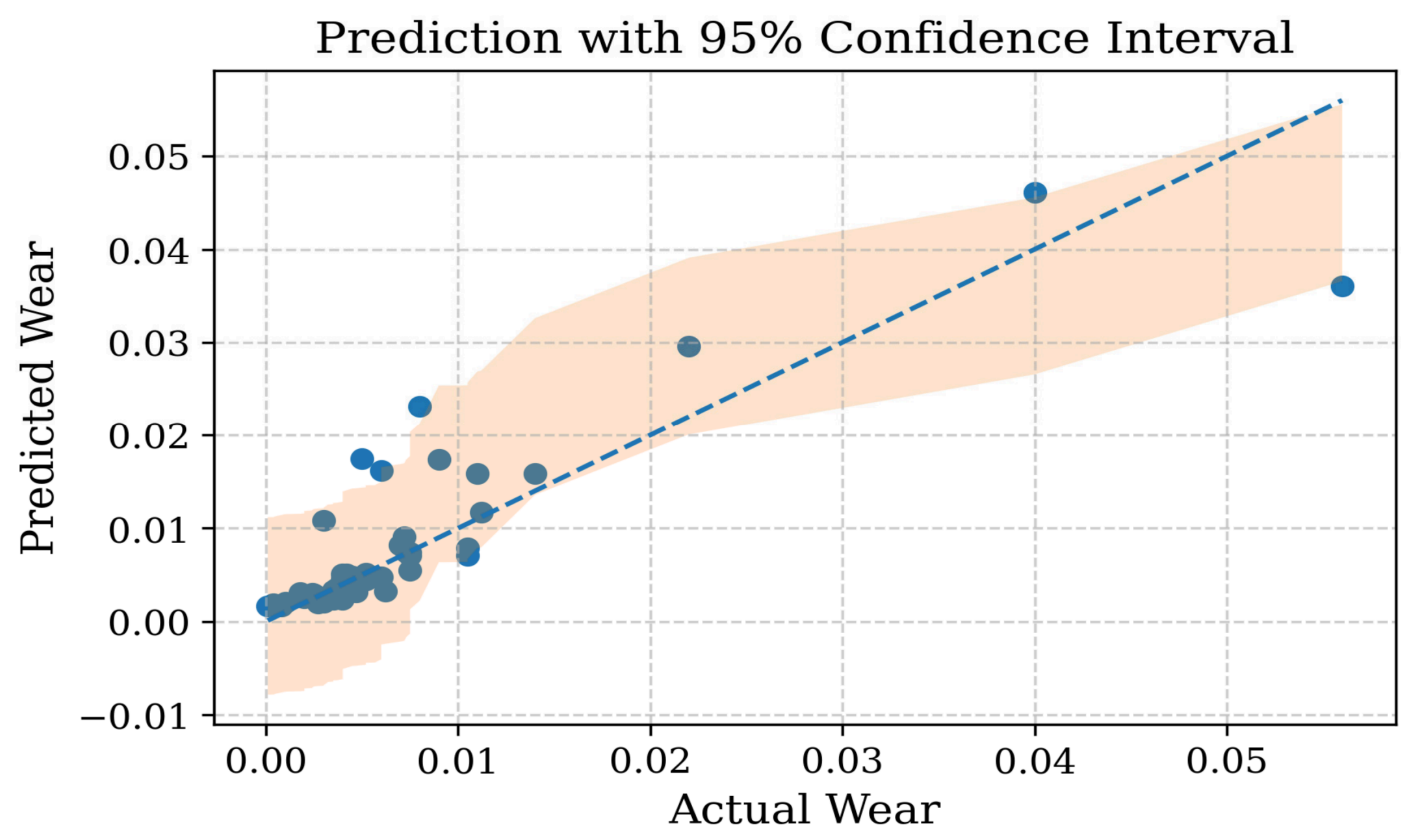
Prediction with 95% confidence interval.

**Figure 18:**
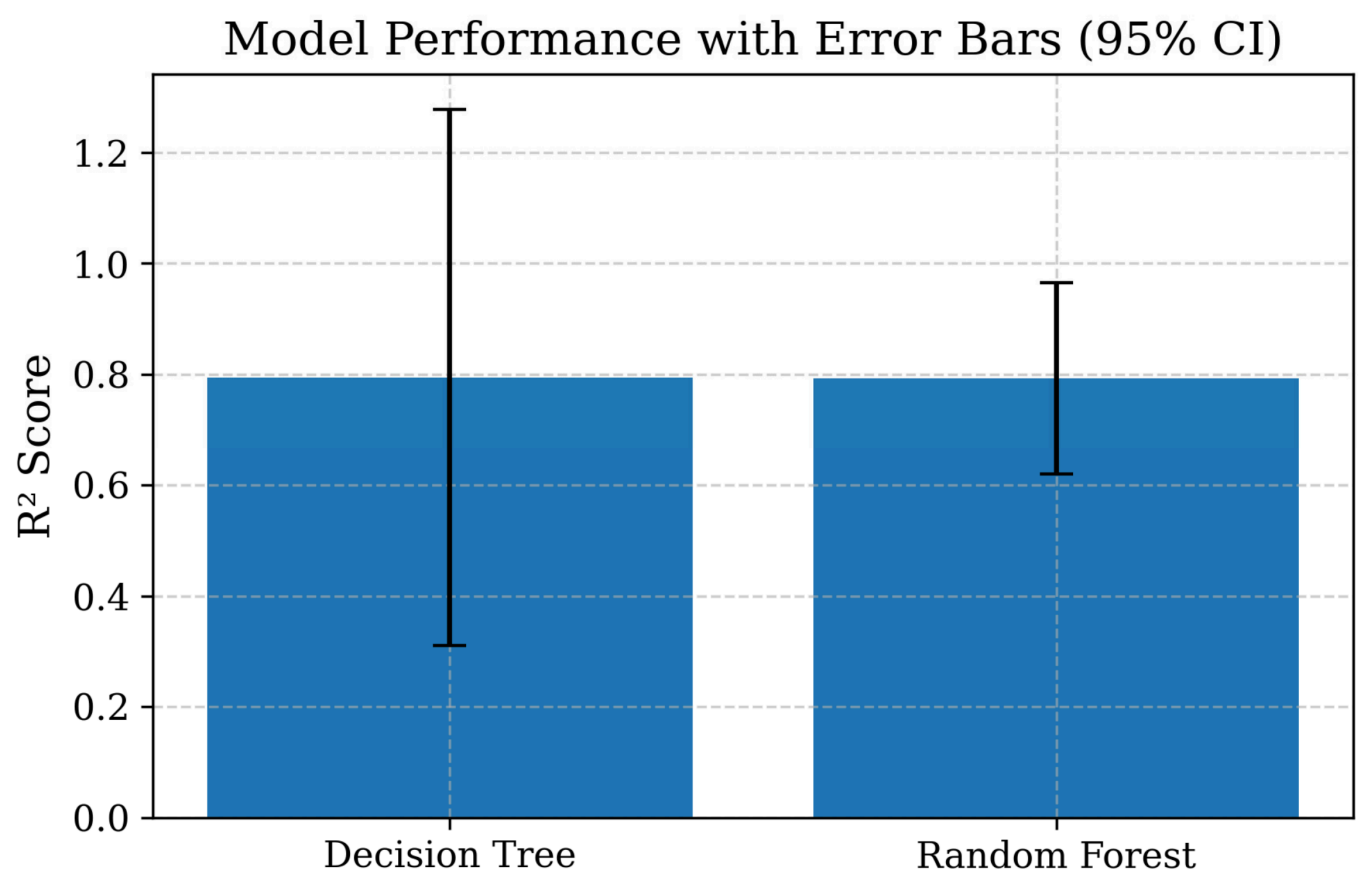
Model performance with error bars.

**Table 1:** Chemical composition of the various steels in as-received condition.

Steel	C (%)	Si (%)	Mn (%)	P (%)	S (%)	Cr (%)	Ni (%)	Total
EN3	0.184	0.179	0.679	0.021	0.004	—	—	98.933
20MnCr5	0.198	0.242	1.124	0.018	0.007	1.025	—	97.386
EN353	0.184	0.244	0.624	0.021	0.022	0.820	1.002	97.083

**Table 2:** As-received condition hardness values.

Steel	Average Hardness (HV)
EN3	151.57
20MnCr5	229.36
EN353	232.73

**Table 3:** Hardness values after the carburization.

Steel	Core	1	2	3	4	5	6	7	Case
EN3	138.54	146.87	131.30	134.64	129.90	130.27	142.67	182.10	215.50
20MnCr5	160.54	177.07	183.77	169.34	175.70	168.97	179.04	249.57	275.74
EN353	159.57	164.10	166.44	164.94	165.44	162.77	168.20	198.70	259.40

**Table 4:** Wear data.

Exp No.	Speed in RPM	Load in kg	Time in min	Wt. Loss (g)					
				EN3 Steel		20MnCr5 Steel		EN353 Steel	
				As Received	Carburized	As Received	Carburized	As Received	Carburized
1	200	1.5	10	0.022	0.003	0.002	0.002	0.002	0.002
2	200	1.5	20	0.035	0.0048	0.0045	0.004	0.0035	0.0034
3	200	1.5	30	0.056	0.0075	0.0073	0.0062	0.0065	0.0051
4	200	3	10	0.024	0.0032	0.0027	0.0024	0.0015	0.0027
5	200	3	20	0.029	0.0052	0.0052	0.0047	0.003	0.004
6	200	3	30	0.055	0.0078	0.008	0.0085	0.0078	0.0057
7	200	4.5	10	0.038	0.0032	0.0032	0.0022	0.0014	0.0027
8	200	4.5	20	0.048	0.0052	0.0057	0.004	0.0018	0.0041
9	200	4.5	30	0.078	0.0078	0.0085	0.007	0.005	0.006
10	400	1.5	10	0.011	0.003	0.0035	0.0025	0.0055	0.0014
11	400	1.5	20	0.018	0.0028	0.006	0.0049	0.009	0.002
12	400	1.5	30	0.03	0.0055	0.009	0.0065	0.0118	0.0034
13	400	3	10	0.014	0.0012	0.0042	0.003	0.0068	0.0011
14	400	3	20	0.019	0.0032	0.0067	0.004	0.0102	0.0024
15	400	3	30	0.026	0.006	0.0095	0.007	0.015	0.004
16	400	4.5	10	0.008	0.001	0.0047	0.0035	0.002	0.001
17	400	4.5	20	0.022	0.0032	0.0072	0.0052	0.0072	0.0024
18	400	4.5	30	0.043	0.0058	0.01	0.0075	0.0102	0.0041
19	600	1.5	10	0.003	0.0015	0.005	0.003	0.0035	0.0011
20	600	1.5	20	0.006	0.0025	0.0075	0.0075	0.0068	0.0014
21	600	1.5	30	0.023	0.005	0.012	0.0105	0.008	0.003
22	600	3	10	0.004	0.001	0.0057	0.0032	0.005	0.001
23	600	3	20	0.005	0.0028	0.0088	0.0065	0.0082	0.0021
24	600	3	30	0.026	0.0055	0.0122	0.0105	0.0112	0.0037
25	600	4.5	10	0.009	0.0018	0.0062	0.004	0.0018	0.0017
26	600	4.5	20	0.016	0.003	0.009	0.0075	0.007	0.002
27	600	4.5	30	0.04	0.0055	0.0115	0.0095	0.0082	0.0037

**Table 5:** Sensitivity analysis.

Test Size	Model	RMSE	MAE	R^2^
0.1	DT	0.002326	0.001199	0.4236
0.1	RF	0.002733	0.001510	0.2043
0.2	DT	0.002802	0.001670	0.5687
0.2	RF	0.003713	0.002117	0.2428
0.3	DT	0.005650	0.002747	0.6406
0.3	RF	0.004868	0.002668	0.7332
0.4	DT	0.005319	0.002584	0.7114
0.4	RF	0.004363	0.002472	0.8059

**Table 6:** Comparative model analysis.

Model	RMSE	MAE	R^2^
Decision Tree	0.00565	0.00275	0.64058
Random Forest	0.00495	0.00273	0.72439
PINN	0.158723	0.125998	282.617610

## Data Availability

The original contributions presented in this study are included in the article. Further inquiries can be directed to the corresponding author.
